# Native Valve* Streptococcus bovis* Endocarditis and Refractory Transfusion Dependent Iron Deficiency Anaemia Associated with Concomitant Carcinoma of the Colon: A Case Report and Review of the Literature

**DOI:** 10.1155/2016/2670307

**Published:** 2016-01-05

**Authors:** Abdul Azeez Ahamed Riyaaz, Randula Samarasinghe, Kolitha Sellahewa, Sabaratnam Sivakumaran, Manjula Sri Tampoe

**Affiliations:** ^1^Department of Internal Medicine, Dr Neville Fernando Teaching Hospital, Millennium Drive, Chandrika Kumaratunga Mawatha, 10115 Malabe, Sri Lanka; ^2^South Asian Institute of Technology and Medicine, Millennium Drive, Chandrika Kumaratunga Mawatha, 10115 Malabe, Sri Lanka

## Abstract

*Streptococcus bovis* is found as a commensal organism in human gut and may become opportunistically pathogenic. Infective endocarditis is one of the commonest modes of presentation of this infection. The association between* Streptococcus bovis* endocarditis and colorectal cancer is well recognized. We report a case of* Streptococcus bovis* endocarditis along with a refractory iron deficiency anaemia associated with concomitant carcinoma of ascending colon in a 63-year-old male. Cooccurrence of these two conditions may cause a challenge in the management. Considering the strong association of colon cancer with* Streptococcus bovis* endocarditis, a detailed screening colonoscopy is mandatory following the diagnosis of the latter.

## 1. Introduction

The gastrointestinal tract in 2.5 to 15% of human population is colonized by* Streptococcus bovis*. Bacteremia and endocarditis are the commonest presentation of infection by this organism when they become pathogenic.* Streptococcus bovis* accounts for around 13% of all infective endocarditis cases and usually causes severe valve damage. The association of* Streptococcus bovis* and gastrointestinal pathologies, colon cancer in particular, has been well established. The aim of this report is to highlight the clinical, diagnostic, and management aspects of a patient who presented with* Streptococcus bovis* endocarditis and a refractory iron deficiency anaemia followed by the detection of a concomitant colon cancer.

## 2. Case Presentation

A 63-year-old man from western part of Sri Lanka with a long-standing history of type 2 diabetes mellitus and hypertension admitted to our medical unit with an acute febrile illness with duration of 2 days. He complained of generalized malaise, anorexia, and weight loss over the last 4 months. Upon systemic inquiry, he did not have any symptoms related to respiratory, gastrointestinal, and genitourinary systems.

He had been under investigation for symptomatic iron deficiency anaemia with no identified cause over the last 4 months which required 2 hospital admissions and 8 blood transfusions. An upper gastroscopy was normal and he was advised to undergo a colonoscopy during his last hospital admission 2 months ago but he refused the procedure. He was discharged and was on oral iron supplements until his current hospital admission.

On examination, he was looking ill and febrile (temperature: 38.3°C). There was severe pallor of his conjunctivae without icterus, lymphadenopathy, skin rashes, or peripheral stigmata of infective endocarditis. Cardiovascular examination revealed a pulse rate of 96 beats per minute, blood pressure of 140/90 mm Hg, a normal JVP, slightly deviated apex beat, and normal 1st and 2nd heart sounds without any additional heart sounds or murmurs. Respiratory system examination was unremarkable and his respiratory rate was 18 breaths per minute. There was an asymmetrical swelling of the right side of his abdomen, deep palpation of which revealed a 10 cm sized nontender, smooth, and rounded mass in the right upper quadrant.

**Figure 1:**
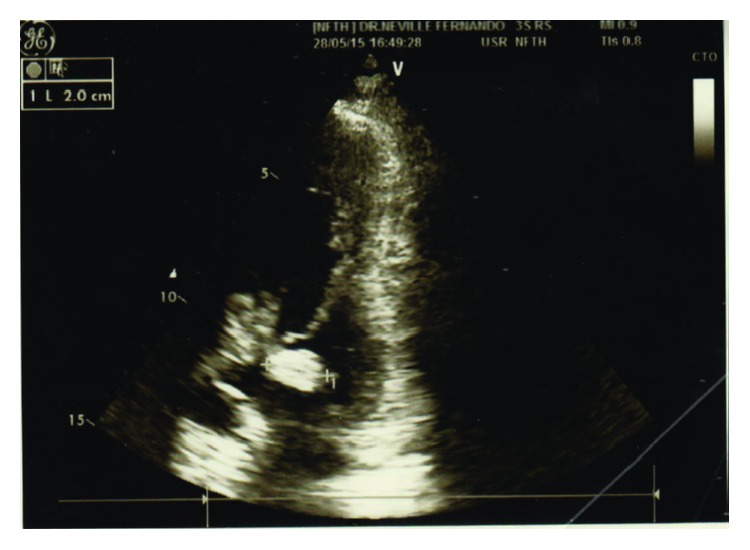
2D echocardiogram showing large sessile vegetation attached to anterior mitral valve leaflet.

**Figure 2:**
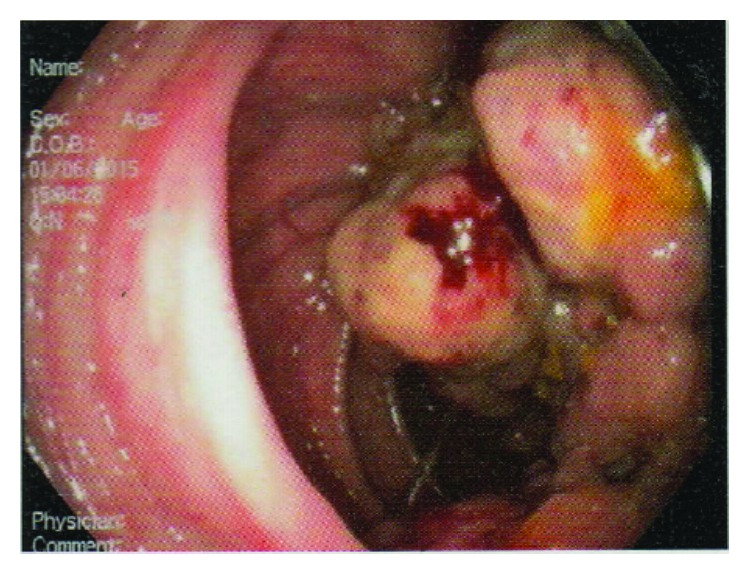
Colonoscopy image of the ascending colon at the level of hepatic flexure showing the tumour.

Initial investigations showed haemoglobin of 5.9 g/dL, white cell count of 9.22 k/mm^3^ with 63% neutrophils, platelets of 360 k/mm^3^, C-reactive protein of 277 mg/dL, normal renal and liver function tests, and a normal chest X-ray. A blood culture taken on admission grew group D* Streptococcus* species after 24 hours of incubation which was identified as* Streptococcus bovis* subsequently. He was started on intravenous Ceftriaxone 2 g daily. On the 3rd day of his hospital admission, he developed a sudden onset left sided hemiparesis and dysarthria which resolved completely within few hours. A noncontrast CT brain did not show any infarction or haemorrhage. Transient ischaemic attack due to possible septic emboli was suspected and 2D echocardiogram was requested which showed a large (18 mm) sessile vegetation attached to the anterior mitral valve leaflet (Figure 1) associated with grade 2 mitral regurgitation and normal left ventricular systolic function. Following the diagnosis of* Streptococcus bovis* endocarditis antibiotic was changed to intravenous benzyl penicillin 4 mu every 6 hours and gentamycin 1 mg/kg every 8 hours. An Abdominal CT scan was done in view of right sided abdominal mass which showed the mass was arising from the ascending colon near hepatic flexure with no metastases to the liver. The patient underwent a colonoscopy which confirmed the growth at the level of hepatic flexure (Figure 2), biopsy of which revealed an adenocarcinoma. His fever settled after the 5th day of antibiotic therapy with serial reduction in inflammatory markers indicating resolution of endocarditis. But he developed frank bleeding per rectum and progressive increase in blood transfusion requirements during the 3rd week of his illness. After a multidisciplinary team discussion involving gastroenterology surgeon, oncologist, and cardiothoracic surgeon it was decided to proceed with an urgent surgery to remove the tumour due to active bleeding although a cardiothoracic surgery too was indicated at that time due to persistent large vegetation and thromboembolic risk. He underwent an extended right hemicolectomy at the end of 5th week of his illness. It was a large tumour arising from the caecum and extending up to hepatic flexure with a staging of T_3_N_0_M_0_ at surgery, biopsy of which revealed a moderately differentiated adenocarcinoma. He had an uneventful recovery and was closely observed and followed up for thromboembolic complication from the vegetation. Follow-up echocardiogram revealed a healed and slightly calcified vegetation with moderate mitral regurgitation without significant left ventricular dysfunction. He is currently awaiting a mitral valve replacement surgery.

## 3. Discussion

*Streptococcus bovis* is found as a commensal organism in 2.5–15% of normal population [1]. It is nonenterococcal Lancefield group D* Streptococcus* for which a new nomenclature exists (Table 1) [2].* S. bovis* is the second commonest streptococcal species to cause infective endocarditis and is responsible for around 13% of all infective endocarditis cases [3]. The portal of entry for this organism is usually the gastrointestinal tract and less commonly the biliary and urinary tracts. Although previous literature reported high prevalence of simultaneous multivalvular involvement in* S. bovis* endocarditis, a very recent single center study shows the prevalence is only around 28%. Our patient had a single valve involvement with a large vegetation and mild valve damage [4]. Higher incidence of neurological complications occurs with* S. bovis* endocarditis and the incidence of stroke is reported to be around 6% [5]. Our patient also developed a transient ischaemic attack possibly due to a septic embolism from the vegetation.

The association between streptococcal endocarditis including* S. bovis* and colon cancer was first described in early 1950s by McCoy and Mason [6]. After 2 decades, Hoppes and Lerner reported 14 cases of* S. bovis* endocarditis in which 9 (64%) cases were associated with concomitant gastrointestinal disease [7]. This association was further strengthened by the findings that the faecal carriage rate for this* S. bovis* was 5 times higher in patients with colonic malignancy compared with normal population and around half of patients with colon cancer develop silent infection with* S. bovis* [8]. In recent systematic review and meta-analysis authors report it is* Streptococcus bovis* biotype I (*Streptococcus gallolyticus*) which has the unambiguous association with gastrointestinal disease compared to other subspecies [9]. The reported incidence of colon cancer in patients with* S. bovis* endocarditis varies between 18 and 62% [10]. Other gastrointestinal pathologies reported with this association include benign colonic lesions, gastric cancer, peptic ulcer disease, inflammatory bowel disease, and liver cirrhosis [11].

It has been controversial whether the association between* S. bovis* bacteremia and endocarditis with colon cancer is a consequence of gastroenterological lesion or it could be related to its aetiopathogenesis. A recent review of research in this field shows this association is aetiological in nature. Cytokines such as IL-1, IFN-gamma, and IL-8, produced by* S. bovis* infection, are most probably responsible for a slow progressing carcinogenesis of colorectal mucosal tissues which appears to occur through a transformation process from normal tissues to premalignant lesions and finally to malignant tissues [12]. Proinflammatory potentials of* S. bovis* including the leucocytic recruitment, the tumor tissue-selective adhesion potential, the selective colonization in tumor cells, the suitable microenvironment of tumor tissues for* S. bovis* proliferation, and the local disruption of tumor tissues and capillaries which allow the entry of* S. bovis* into blood circulation seem to contribute to bacteremia [12].

The management of* S. bovis* endocarditis is straightforward as in other streptococcal endocarditis once the diagnosis is made. Emergency or urgent valve replacement surgery may be indicated in many cases since the incidence of haemodynamically unstable valve damage and neurological complications are common with* S. bovis* endocarditis. Management dilemma may arise as in our patient if there is concomitant colon cancer which warrants urgent surgery particularly when both bowel and cardiothoracic surgery are indicated in relation to preference and timing of surgery. We could not find any publications or studies addressing these issues in our literature search. Thus it is extremely useful to involve multidisciplinary team including gastroenterology surgeon, oncologist, and cardiothoracic surgeon in the decision-making process.

## 4. Conclusions

Considering the strong association between* S. bovis* septicaemia and/or endocarditis and colonic carcinoma it cannot be overemphasized that a detailed colonoscopy is mandatory in the assessment of these patients. Moreover, unexplained iron deficiency anaemia as in our patient is an additional indication for colonoscopy as it is a well-recognized presentation of occult right sided colonic malignancy. Further research is needed to address the management issues when* S. bovis* endocarditis and colon malignancy coexist.

## Figures and Tables

**Table 1:** Nomenclature of the principal human species of *Streptococcus bovis*.

Old nomenclature	Recent nomenclature
*Streptococcus bovis* biotype I	*S. gallolyticus *subsp.* gallolyticus*
*Streptococcus bovis* biotype II/1	*S. infantarius *subsp.* infantarius* *S. infantarius *subsp.* coli*
*Streptococcus bovis* biotype II/2	*S. gallolyticus *subsp.* pasteurianus*

## Abbreviations

JVP: Jugular venous pressure
CT: Computerized tomography
*S. bovis*: * Streptococcus bovis*
IL: Interleukin
IFN: Interferon.
